# Air entrapment resembling necrotising fasciitis as a frequent incident following total hip arthroplasty

**DOI:** 10.1038/s41598-019-52113-9

**Published:** 2019-10-31

**Authors:** Maria A. Smolle, Nina Hörlesberger, Ewald Musser, Michael Maier, Patrick Reinbacher, Jörg Friesenbichler, Andreas Leithner, Werner Maurer-Ertl

**Affiliations:** Department of Orthopaedics and Trauma, Medical University of Graz, Graz, Austria

**Keywords:** Orthopaedics, Outcomes research

## Abstract

In combination with pain and elevated inflammatory parameters that are frequently observed following elective total hip arthroplasty (THA), air entrapment on radiographic images could be indicative of necrotising fasciitis. The aim of the present study was to analyse presence/extent of air entrapment following THA, and to correlate radiological with clinical findings. One-hundred patients undergoing short-stem elective THA (ANA NOVA Alpha Proxy-system) were prospectively included. Patients received pre- and postoperative x-rays (day 1 + discharge) of the affected hip, together with a CT-scan of the lower extremity (discharge). C-reactive-protein-(CRP), leukocyte, haemoglobin-, creatinine-, glucose-, sodium-levels – and based on these the *LRINEC* score– as well as pain-scores (numeral-rating-scale, NRS) at postoperative days 1, 3 and 5 were documented. Air entrapment was visible in 98% of x-rays taken postoperatively and in 93% of CT-scans at discharge. Leukocyte-levels significantly decreased from postoperative day 1 to 5. CRP-levels had a peak at the 3^rd^ postoperative day (p < 0.001). On discharge-x-rays of patients with low body-mass-indexes, air entrapment was significantly more often visible (p = 0.040). Neither implant-related nor laboratory parameters, *LRINEC*- or NRS-scores significantly correlated with presence/extent of air entrapment (p > 0.05). Considering the high rate of air entrapment following elective THA postoperatively and at discharge, suspicion of an infection with gas-producing bacteria may only be raised in case of persistent inflammatory parameters, deteriorating general condition and signs of local infection.

## Introduction

Severe pain following elective total hip arthroplasty (THA) is uncommon as patients are usually treated according to the *World Health Organisation’s* (WHO) three-step-ladder for pain management^1,2^. In some cases, however, pain persists over several days, wherefore further investigative procedures as plain x-rays and CT-scans may be performed in order to rule out sintering of the stem or even periprosthetic fracture^2,3^. In case air entrapment is visible on these scans, wound infection with gas-producing bacteria (e.g. clostridium perfringens) may be considered as differential diagnosis for inexplicable pain. As a result of this suspected life-threatening condition also known as necrotising fasciitis, immediate surgical revision, antibiosis and intensive care would become necessary^4–7^.

There have been some case reports on air entrapment following orthopaedic or trauma surgeries with implants where presence of any infection could be subsequently ruled out^8–10^. Case-series investigating presence of air entrapment following THA are currently missing, though.

Therefore, the aim of the present study was to 1) analyse the presence of air entrapment following THA directly postoperatively on X-rays and follow-up X-rays as well as CT-scans before discharge, and to 2) evaluate which factors correlate with presence of air entrapment.

To the best of our knowledge, this is the first systematic study investigating presence and extent of air entrapment following elective THA.

## Results

### Descriptive analysis

**Figure 1:**
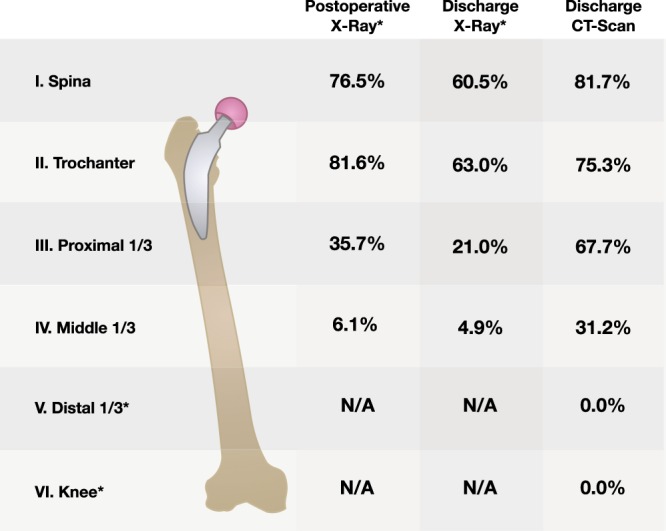
Air entrapment. Frequency (in %) of air entrapment in x-rays taken directly postoperatively and x-rays as well as CT-scans taken at discharge. *X-rays did only cover the region up to the middle 1/3 of femur, whilst the CT-scan covered all regions.

In 98% of cases, air entrapment was visible at the initial postoperative x-ray, taken at day of surgery in 90 patients, one day following surgery in 9 patients and two days following THA in 1 patient. In most cases, 2 regions were affected by air entrapment (48.5%), followed by 3 (26.3%) and 1 region (21.2%). Distribution of regions is visible in Fig. 1 for the x-rays taken postoperatively and at discharge as well as for the CT-scan at discharge.

**Figure 2:**
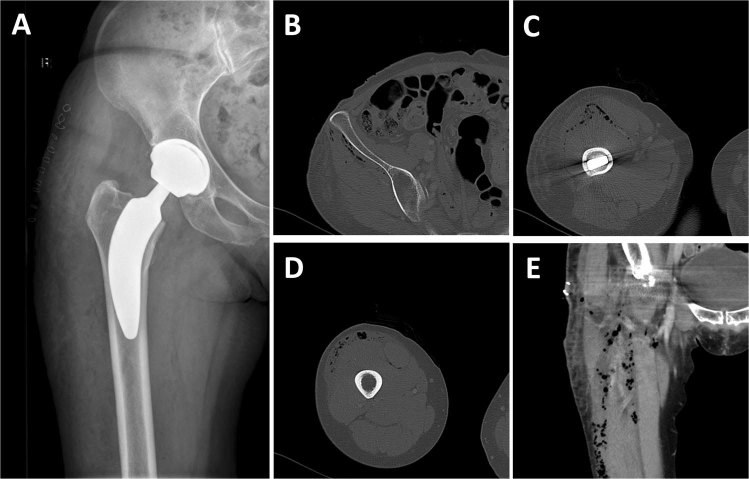
Clinical case. X-ray taken directly postoperatively from a 57-year-old female patient who had undergone THA (ANA NOVA Alpha 50/44/36 M/Proxy 8) of the right hip. (**A**) X-ray of the left hip taken 1 day postoperatively (**B**) and corresponding CT-scan (**C**). Note the air distribution from the anterior superior iliac spine (**B**) down to the proximal 1/3 (**C**) and mid 1/3 of the femur (**D**,**E**).

In 17 cases of x-rays taken at discharge after a median of 3 days (interquartile range [IQR]: 3–3 days) postoperatively, the air entrapment could no more be detected. On the contrary, air entrapment was still present in 82.7% of cases (Fig. 2). Of note, in 43.3% of cases, only one region was affected by air entrapment at discharge, indicating air resorption over time.

CT-scans taken after a median of 3 days (IQR: 3–3 days) following surgery showed a slightly different pattern, due to the higher accuracy of this imaging technique: Air entrapment was still present in 93% of cases, with three regions being affected in 40.4% of cases, followed by two regions (20.2%). Of note, in a considerable number of cases (n = 63), air entrapment at the proximal 1/3 of femur could be demonstrated, which had been invisible in most postoperative x-rays. Moreover, air entrapment at the mid-shaft of femur was detectable in 29 cases. Neither in the postoperative x-rays, nor the x-rays and CT-scans taken upon discharge, any air entrapment in the distal 1/3 of femur or knee region could be detected.

**Figure 3:**
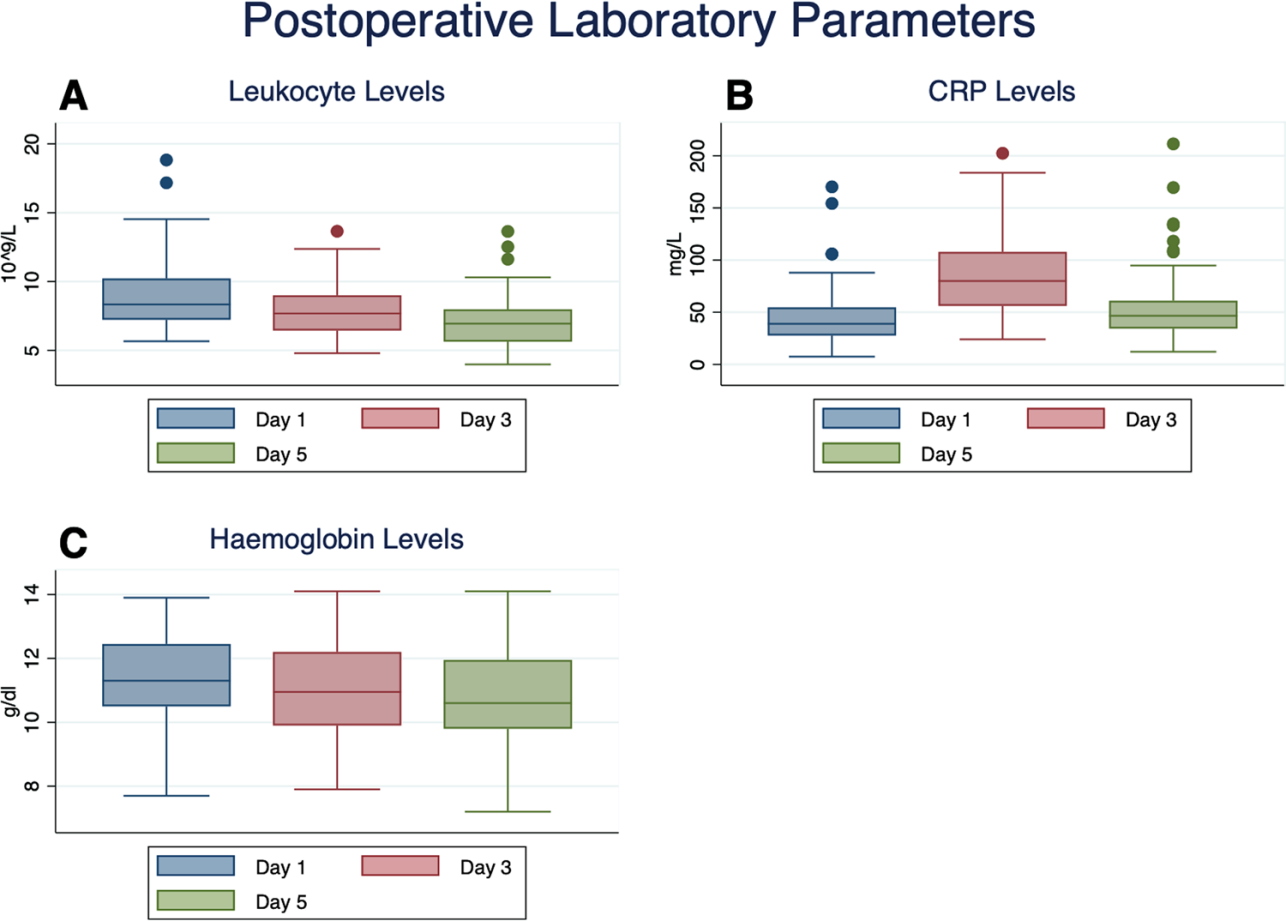
Postoperative laboratory parameters. Postoperative leukocyte levels at days 1, 3 and 5. (**A**) The constant drop was statistically significant for all three timepoints (paired t-test; 1 vs. 3: p < 0.001; 3 vs. 5: p < 0.001; 1 vs. 5: p < 0.001). CRP levels at postoperative days 1, 3 and 5, with a peak observed at day 3. (**B**) All changes were statistically significant (paired t-test; 1 vs. 3: p < 0.001; 3 vs. 5: p < 0.001; 1 vs. 5: p = 0.017). Haemoglobin levels at days 1, 3 and 5 postoperatively. (**C**) There was a significant drop between days 1 and 3 and 1 and 5, whilst there was no significant drop from day 3 to 5 (paired t-test; 1 vs. 3: p < 0.001; 3 vs. 5: p = 0.985; 1 vs. 5: p < 0.001).

Mean leukocyte levels were 8.99, 7.87 and 7.12 10^9/L at postoperative days 1, 3 and 5, respectively (normal range: 4.4–11.3 10^9/L; Fig. 3A). For the same time-point, mean CRP-levels were 45.7, 86.4 and 55.1 mg/L (normal range: 0–5 mg/L; Fig. 3B) and mean haemoglobin-levels 11.4, 10.9 and 10.8 g/dl (normal range: 12.0–15 g/dl; Fig. 3C). Mean sodium-, glucose and creatinine-levels at the first postoperative day were 139 mmol/L (normal range: 135–145 mmol/L), 134 g/dl (normal range: 70–100 mg/dl) and 0.89 mg/dl (normal range: < 1 mg/dl). NRS-scores as measured at days 1, 3 and 5 ranged between 0 and 5 (mean: 1.1), 0 and 4 (mean: 0.3) and 0 and 4 (mean: 0.3), respectively. They significantly decreased between days 1 and 5 (paired t-test; p < 0.001). Wound healing was unremarkable in all cases, without any signs of local infection and secretion having ceased at the first or second change of dressings.

### Correlation with clinical parameters

Neither time of surgery (t-test; p = 0.676, p = 0.995, p = 0.540), patient’s age (t-test; p = 0.945, p = 0.341, p = 0.261), shaft size (t-test; p = 0.663, p = 0.091, p = 0.597), head size (t-test; p = 0.722, p = 0.526, p = 0.251) nor cup size (t-test; p = 0.425, p = 0.626, p = 0.747) did significantly correlate with presence of air entrapment in postoperative X-ray, postoperative CT-scan or X-ray at discharge, respectively. Air entrapment was more often visible on x-rays taken at discharge in patients with a low body mass index (BMI; 28.1 vs. 30.7; t-test; p = 0.040), whilst there was no difference concerning BMI for X-rays taken directly postoperatively (t-test; p = 0.430) or CT-scans taken upon discharge (t-test; p = 0.908). NRS-scores at days 1 and 5 were not significantly correlated with presence of air entrapment (chi-squared-test; Table 1). At postoperative day 3, however, the proportion of patients with NRS-scores >2 was smaller in case air entrapment was present on postoperative CT-scans (chi-squared-test, p = 0.011; Table 1).

**Table 1:** Parameters associated with air entrapment.

Air Entrapment		Day 1 Postoperative X-Ray			Day 3 Postoperative CT			Day 5 X-Ray at Discharge		
		No	Yes	p-value	No	Yes	p-value	No	Yes	p-value
*Leukocytes 10^9*		6.92	9.03	0.225	7.29	7.85	0.469	7.76	6.94	0.167
*CRP mg/l*		42.6	45.7	N/A	76.9	85.0	0.608	51.4	53.6	0.844
*Haemoglobin g/dl*		11.1	11.4	0.771	11.3	10.9	0.542	11.0	10.8	0.617
*LRINEC (n)*	***≤5***	N/A			6	89	0.604	17	79	0.513
	***> 5***				0	4		0	2	
*NRS (n)*	***0–2***	2	78	0.510	5	86	**0.011**	8	55	0.703
	***≥ 3***	0	17		1	1		0	1	

Comparison of laboratory parameters, LRINEC- and NRS-scores with presence of air entrapment at postoperative days 1, 3 and 5. *N/A – not applicable (due to low case number)*

### Correlation with laboratory parameters & NRS-scores

There was no significant correlation between presence of air entrapment and CRP-, leukocyte-, haemoglobin-levels at days 1, 3 or 5 postoperatively (t-test; p > 0.05; Table 1). *Laboratory risk indicator for necrotizing fasciitis* (LRINEC) scores did not correlate with presence of air entrapment (Table 1). However, whilst on postoperative day 1, none of the patients presented with a LRINEC score >5, on postoperative days 3 and 5, four and two patients, respectively, had a LRINEC score >5. In each case, air entrapment had been present on postoperative CT or X-ray at discharge, whilst NRS-scores were ≤ 1.

## Discussion

In the present study evaluating the presence of air entrapment following elective total hip arthroplasty, this phenomenon was visible in 98% of x-rays taken directly postoperatively. Upon discharge, air entrapment was still present in 82.7% of x-rays, whilst residual air could be proven in even 93% of CT-scans taken after a median of 3 days following surgery. The only factor being significantly associated with the presence of air entrapment on the x-ray taken at discharge was a low BMI, whilst clinical, implant-related and laboratory parameters showed no significant association.

Several theories about causes for air entrapment following orthopaedic or trauma surgery with implants have been raised in the past: Reaming of the bone during surgery may cause efflux of intramedullary fat, blood, and air into the surrounding soft tissues, leading to emphysema^8^. This theory may apply for minimally-invasive implanted devices as femoral, tibial and humeral nails. In the present cohort, however, reamers were not used.

In a single case-report, pneumarthrosis of the knee had been observed following arthroscopy in a patient with several previous surgeries at the same site^11^. The authors hypothesised that scarred tissue had lost its mechanical ability to “seal” the wound from air influx^11^. In view of the fact that our patients underwent the very first surgical procedure at the respective extremity, this theory seems rather inapplicable to the present cohort.

An alternative mechanism causing air entrapment within muscles remote from the actual surgical site could be by mechanical traction. Due to the fact that considerable passive movements of the affected extremity are required during THA via an anterolateral approach (e.g. quadripartite-position), air may be conveyed in between muscular septa. This could explain why in 63 and 29 cases air entrapment in the proximal and mid 1/3 of femur, respectively, could be detected.

There have been only two case-reports up to date describing necrotising fasciitis following THA^12,13^. In the case reported by *El-Karef et al*., a 75-year old female patient started to feel unwell at postoperative day 5 and presented with fever (39 °C), elevated leukocyte-levels (16.0 10^9/L) and local erythema with central necrosis^12^. Following intravenous antibiotics, the patient deteriorated again and the ulcerated lesion grew rapidly. Ultimately, the affected tissue was resected en-bloc, followed by plastic reconstruction to cover the extensive soft-tissue defect^12^.

In the second case report by *Sharma et al*., a 71-year-old woman presented with sudden onset of fever (38.5 °C) and a painful right hip following THA ten days earlier^13^. Leukocyte-levels were distinctly elevated (33.9 10°9/L), as were CRP-levels (366 mg/L). The wound was surrounded by erythema, lysis of the epidermis and a rapidly advancing necrotic area^13^. Also, in this case, initial aggressive treatment with intravenous antibiotics failed, and extensive, repeated surgical debridement became necessary. As further radiographs and swab culture samples confirmed deep infection, the prosthesis had to be explanted and an antibiotic-loaded cement spacer was implanted. Ultimately, the patient underwent a Girdlestone excision arthroplasty^13^.

None of our patients actually had a necrotising fasciitis, which in CT-imaging would very likely have shown not only air-entrapment but also thickening of fascia, subcutaneous emphysema, fluid collections and non-enhancing fascia indicating the presence of necrosis^14,15^. These features, however, may also be imitated by the postoperative situation. The typical signs such as local erythema and tenderness, swelling, pain hardly responsive to analgesics as well as a deteriorating general condition with tachycardia, hypotension, fever and tachypnoea, were also absent in our collective^12,13^. On the other hand, laboratory parameters and especially infection scores (i.e. LRINEC score^16^) alone were not very useful in ruling out necrotising fasciitis following THA, though leukocytosis, hyponatraemia and elevated C-reactive protein (CRP) have been suggested as valuable predictors^16^. CRP levels may become relatively high following elective THA; in our cohort, a peak at day 3 postoperatively was observed, with mean CRP levels of 86.4 mg/L, that had subsequently decreased at day 5. Conversely, leukocyte levels constantly decreased from postoperative days 1 to 5 and did not exceed normal ranges at any time. In addition, NRS-scores were low, ranging between 1 and 5 at the first postoperative day, whilst having significantly decreased by day 5. Bearing the conventional laboratory course following elective THA in mind, elevated CRP-levels in combination with low haemoglobin-levels, unremarkable leukocyte-levels and low NRS scores would unlikely be indicative of underlying necrotising fasciitis.

When assessing the LRINEC score as proposed by *Wong et al*. to evaluate the risk of necrotising fasciitis being present, four patients presented with a LRINEC score of 6 and air entrapment on CT-scans at postoperative day 3. Interestingly, a LRINEC score >5 is considered as “intermediate” for necrotising fasciitis being present. In or cohort, however, none of the patients actually developed this life-threatening disease, despite air entrapment and “intermediate” LRINEC scores. Notably, NRS-scores were ≤1 in those patients with LRINEC scores >5.

There is currently no clear explanation why a low BMI was associated with the presence of air entrapment upon discharge. Potentially, intermuscular air is better visible in case subcutaneous tissue is scarce, wherefore small pneumatic areas may not be contrasted on plain radiographs in obese patients, whilst they are still traceable on CT-scans.

Considering the high-rate of air entrapment as seen in elective total hip arthroplasty directly postoperatively and still at discharge, the suspicion of infection with gas-producing bacteria may only be raised in case of radiological air entrapment in combination with severe pain, local signs of infection and deteriorating patients’ condition, whilst high CRP- as well as reduced haemoglobin-levels are rather the consequence of surgery than an indication of underlying necrotising fasciitis.

## Material and Methods

One-hundred patients undergoing elective THA between February 2016 and March 2017 at one orthopaedic department were prospectively included. All patients received an ANA NOVA Alpha Cup (*ImplanTec GmbH, Moedling, Austria*) and an ANA NOVA Alpha Shaft Proxy ( = short stem; *ImplanTec GmbH, Moedling, Austria*). All patients had undergone surgery by the same orthopaedic surgeon via an anterolateral approach to the hip. Retractors were positioned in a standardised manner. Wounds were closed with sutures of the fascia and the subcutaneous tissue, as well as staples for the skin. Preoperatively, all patients received a standardised antibiotic prophylaxis as single-shot (2^nd^ generation cephalosporins or in case of penicillin-allergy clindamycin). Patients were mobilised the day after surgery with two crutches and full-weight bearing. Pain management was performed according to the WHO’s three-step ladder. Every two days postoperatively, change of dressings was performed to allow for evaluation of the wound situation.

Every patient had a preoperative x-ray of the pelvis and affected hip, followed by a postoperative X-ray at day of surgery or one day afterwards and an X-ray at discharge together with a low-dose CT-scan of the entire lower extremity (pelvis, knee, foot). This CT-scan had primarily been performed to evaluate changes in the anatomy following implantation of the novel short stem-system.

The postoperative x-ray, the x-ray at discharge and the CT-scan at discharge were scanned for air entrapment. Location of entrapment was documented for 6 different regions: anterior superior iliac spine, trochanter, proximal 1/3 of femur, mid 1/3 of femur, distal 1/3 of femur and knee. Additionally, C-reactive-protein (CRP)-, leukocyte- and haemoglobin-levels as measured on days 1, 3 and 5 postoperatively were documented, together with sodium-, creatinine- and glucose-levels usually ascertained at the first postoperative day only. These parameters were used to assess the *Laboratory risk indicator for necrotizing fasciitis* (LRINEC) score at postoperative days 1, 3 and 5 (Supplementary Table 1). Moreover, the numeral rating scale (NRS) scores at days 1, 3 and 5 in the morning at rest were ascertained. Patients were discharged after a median of 6 days (IQR: 5–7 days) with dry wounds, no signs of local infection, ambulatory with crutches or wheel walkers and fully weight-bearing the affected lower limb.

All patients had an unremarkable postoperative follow-up and at a mean follow-up of 1.9 years following surgery (range: 1.0–3.0 years), no periprosthetic fracture, local infection, implant failure or aseptic loosening had been observed. All but one patient, who died from the present cohort due to intracranial haemorrhage, were still alive at last follow-up.

All methods were carried out in accordance with relevant guidelines and regulations. The present study has been approved by the Institutional Review Board of the *Medical University of Graz, Austria* (IRB-No. 28–152 ex 15/16). A written informed consent was obtained from all study participants prior to enrolment in the study.

### Statistical analysis

Statistical analysis was carried out using Stata Version 15.1 (*StataCorp, College Station, Texas, US*). Chi-squared- and (paired) t-tests were performed. Laboratory parameters and NRS-scores at postoperative days 1, 3 and 5 were compared with presence of air entrapment on postoperative X-rays, postoperative CT-scans and X-rays at discharge. A two-sided p-value of < 0.05 was considered statistically significant.

## Supplementary information

Supplementary Table 1.

## Data Availability

The data that support the findings of this study are available from *Implantec (ImplanTec GmbH, Moedling, Austria)* but restrictions apply to the availability of these data, which were used under license for the current study, and so are not publicly available. Data are however available from the authors upon reasonable request and with permission of *Implantec (ImplanTec GmbH, Moedling, Austria)*.

## References

[1] Gaffney CJ, Pelt CE, Gililland JM, Peters CL (2017). Perioperative Pain Management in Hip and Knee Arthroplasty. Orthop Clin North Am.

[2] Baert IAC (2017). Short stem total hip arthroplasty: Potential explanations for persistent post-surgical thigh pain. Med Hypotheses.

[3] Piscitelli P (2013). Painful prosthesis: approaching the patient with persistent pain following total hip and knee arthroplasty. Clin Cases Miner Bone Metab.

[4] Baer W, Schaller P, Ruf S, Lehn N, Lerch K (2002). Diagnosis and therapy of necrotizing fasciitis. Orthopade.

[5] Juttner FM, Pinter H, Vilits P, Smolle J (1985). Fournier gangrene with involvement of the thigh–radical restoration by exarticulation of the femur. Case report. Urologe A.

[6] Wilson B (1952). Necrotizing fasciitis. Am Surg.

[7] Corona PS (2016). Necrotising fasciitis of the extremities: implementation of new management technologies. Injury.

[8] Leyendecker AG, Nicolai P, Blakemore ME (2001). Surgical emphysema formation during intramedullary reaming of a humerus. Injury.

[9] Kantelberg C, Meyer C, Harland U (2014). Benign subcutaneous emphysema after nail penetration. Case report and important differential diagnoses. Unfallchirurg.

[10] Horlesberger N, Hohenberger G, Matzi V, Grechenig P (2018). Extensive emphysema after intramedullary nailing of a pertrochanteric fracture: Life-threatening infection or benign complication. Unfallchirurg.

[11] Fernyhough J, Razza BE (1992). Tension pneumarthrosis complicating arthroscopy of the knee. Am J Sports Med.

[12] El-Karef E, Tiwari A, Aldam C (2000). Necrotizing fasciitis: a rare complication of total hip replacement. J Arthroplasty.

[13] Sharma H, Kelly MP (2007). Acute near-fatal necrotising fasciitis complicating a primary total hip replacement. J Bone Joint Surg Br.

[14] Markeson D., Nijjar R., Evgeniou E., Kulkarni M. (2012). An elderly patient presenting with hip pain following a fall: an unusual presentation of necrotising fasciitis. Case Reports.

[15] Fayad LM, Carrino JA, Fishman EK (2007). Musculoskeletal infection: role of CT in the emergency department. Radiographics.

[16] Wong CH, Khin LW, Heng KS, Tan KC, Low CO (2004). The LRINEC (Laboratory Risk Indicator for Necrotizing Fasciitis) score: a tool for distinguishing necrotizing fasciitis from other soft tissue infections. Crit Care Med.

