# Supplementary material for: Air entrapment resembling necrotising fasciitis as a frequent incident following total hip arthroplasty
Source: Sci Rep. 2019 Oct 31;9:15766. doi: 10.1038/s41598-019-52113-9 (PMC6823472; doi:10.1038/s41598-019-52113-9)
Supplement: Supplementary file 1 — Supplementary Table 1. [file 41598_2019_52113_MOESM1_ESM.docx]

**Air entrapment resembling necrotising fasciitis as a frequent incident following total hip arthroplasty.**

**Short Title**: Air entrapment in total hip arthroplasty.

MARIA A SMOLLE*^1^; NINA HÖRLESBERGER^1^; EWALD MUSSER^1^, MICHAEL MAIER^1^; PATRICK REINBACHER^1^; JÖRG FRIESENBICHLER^1^, ANDREAS LEITHNER^1^; WERNER MAURER-ERTL^1^

^1^Department of Orthopaedics and Trauma, Medical University of Graz, Austria (MAS: [maria.smolle@medunigraz.at](mailto:maria.smolle@medunigraz.at); NH: [nina.hoerlesberger@aon.at](mailto:nina.hoerlesberger@aon.at) EM: [ewald.musser@medunigraz.at](mailto:ewald.musser@medunigraz.at); MM: [Michael.maier@medunigraz.at](mailto:Michael.maier@medunigraz.at); PR: [reinbacher.patrick@gmail.com](mailto:reinbacher.patrick@gmail.com); JF: [joerg.friesenbichler@medunigraz.at](mailto:joerg.friesenbichler@medunigraz.at) AL: [andreas.leithner@medunigraz.at](mailto:andreas.leithner@medunigraz.at); WME: [werner.maurer-ertl@medunigraz.at](mailto:werner.maurer-ertl@medunigraz.at))

**Corresponding Author**

Maria A Smolle, MD

Department of Orthopaedics and Trauma, Medical University of Graz

Auenbruggerplatz 5, 8036 Graz, Austria

E-Mail: [maria.smolle@medunigraz.at](mailto:maria.smolle@medunigraz.at)

Phone: +43 316 385 81881

Fax: +43 316 385 14806

**Supplementary Material**

**Supplementary Table 1. LRINEC score.** Laboratory risk indicator for necrotising fasciitis (LRINEC), according to *Wong et al*.^16,17^.

| **Supplementary Table 1. LRINEC score.** | | |
| --- | --- | --- |
| **Parameter** | **Level** | **Score** |
| *C-reactive protein* | <150 mg/l | 0 |
|  | > 150 mg/l | 4 |
| *Leukocyte levels* | < 1.5 10^9/L | 0 |
|  | 1.5 – 2.5 10^9/L | 1 |
|  | > 2.5 10^9/L | 2 |
| *Haemoglobin levels* | > 13.5 g/dl | 0 |
|  | 11 – 13.5 g/dl | 1 |
|  | < 11 g/dl | 2 |
| *Glucose levels* | ≤ 180 mg/dl | 0 |
|  | > 180 mg/dl | 2 |
| *Sodium levels* | ≥ 135 mmol/l | 0 |
|  | < 135 mmol/l | 2 |
| *Creatinine levels* | ≤ 1.6 mg/dl | 0 |
|  | > 1.6 mg/dl | 2 |
| **Total Score** | | |
| *Low Risk* | | **≤ 5** |
| *Intermediate Risk* | | **6 – 7** |
| *High risk* | | **≥ 8** |
